# First Case Report of Maturity-Onset Diabetes of the Young Type 4 Pedigree in a Chinese Family

**DOI:** 10.3389/fendo.2019.00406

**Published:** 2019-07-03

**Authors:** Mingqun Deng, Xinhua Xiao, Liyuan Zhou, Tong Wang

**Affiliations:** Key Laboratory of Endocrinology, Department of Endocrinology, Ministry of Health, Peking Union Medical College Hospital, Diabetes Research Center of Chinese Academy of Medical Sciences, Peking Union Medical College, Beijing, China

**Keywords:** MODY4, PDX1, pancreatic exocrine dysfunction, obese, Chinese

## Abstract

Maturity-onset diabetes of the young (MODY) is the most common monogenetic diabetes, which is easily misdiagnosed. We describe the first Chinese MODY4 family with a novel mutation, indicating that MODY4 cannot be excluded in early-onset obese diabetes, and pancreatic exocrine dysfunction could be present in MODY4.

## Introduction

Maturity-onset diabetes of the young (MODY) is caused by autosomal dominant mutations, and 14 MODY subtypes have been identified at present. The correct diagnosis provides accurate genetic counseling and may help with appropriate management. However, the clinical overlap between MODY and type 2 diabetes (T2D) or type 1 diabetes (T1D) makes it difficult to diagnose in an accurate and timely manner.

The US SEARCH for Diabetes in the Youth study reported that most MODY cases were misdiagnosed as type 1 (36%) or type 2 diabetes (51%) (1). Liu et al. suggested that, without genetic diagnosis, the clinical misdiagnosis rate of MODY2 was 85.4% (47/55) in China, and 58.2% (32/55) of MODY2 patients received incorrect treatment (2). MODY1-3 are the most common forms of MODY. MODY4 is a rare type of MODY that is caused by heterozygous PDX1 gene mutations. PDX1 encodes the insulin promoter factor-1 (IPF1), a transcription factor that plays a critical role in pancreatic and beta cell development and function (3). Previous studies of heterozygous carriers of PDX1 mutations suggest that MODY4 is a relatively mild disorder of glucose intolerance characterized by diminished insulin secretion (4–6). In the present report, we describe a 14-year-old Chinese boy who was misdiagnosed with T1D and ultimately diagnosed with MODY4 after genetic analysis. This is the first reported case of MODY4 in China, which indicated that obese MODY4 could be disguised.

## Case Report

The proband was born in 2005 with a birth weight of 3,900 g. His mother denied gestational diabetes mellitus. The proband was diagnosed with type 1 diabetes (T1D) in October 2018, with positive uric ketone and fasting blood glucose (FBG) 20 mmol/L. At diagnosis, his height was 181 cm, and his weight was 80 kg, with a body mass index (BMI) of 24.4 kg/m^2^. He was treated with a total dose of 23 units of insulin per day. He presented to the Department of Metabolism & Endocrinology in Peking Union Medical College Hospital (PUMCH) 1 month after the onset of diabetes. His fasting C-peptide (FCP) was 1.10 ng/ml, and 2 h postprandial C-peptide (2hCP) was 3.36 ng/ml, with FBG 6.4 mmol/l and 2 h postprandial glucose (2hPG) 8.1 mmol/l. Glycated hemoglobin (HbA1c) was 10.7% and glycated albumin (GA%) was 23.5%. The patient was negative for the glutamic acid decarboxylase antibody (GAD-Ab), insulinoma-associated protein-2 antibody (IA2-Ab), and islet cell antibody (ICA). Insulin was discontinued, and he was treated with metformin since then. However, approximately half a month later, he experienced occasional hypoglycemia with only metformin 0.5 g QD. FBG was 5.3 mmol/L, and 2hPG 5.1 was mmol/L. No oral antidiabetic medications were prescribed for him since December 2018.We learned that he continued to run every day since the diagnosis of diabetes. His weight was only 63 kg, and his BMI was 18.8 kg/m^2^ when hypoglycemia occurred. However, the proband had occasional abdominal pain since the onset of diabetes. Although no abnormality was revealed by abdominal ultrasound, his serum amylase level was 34 U/L (normal range: 25–115), and his serum lipase level was 63 U/L (normal range: 73–393).

**Figure 1:**
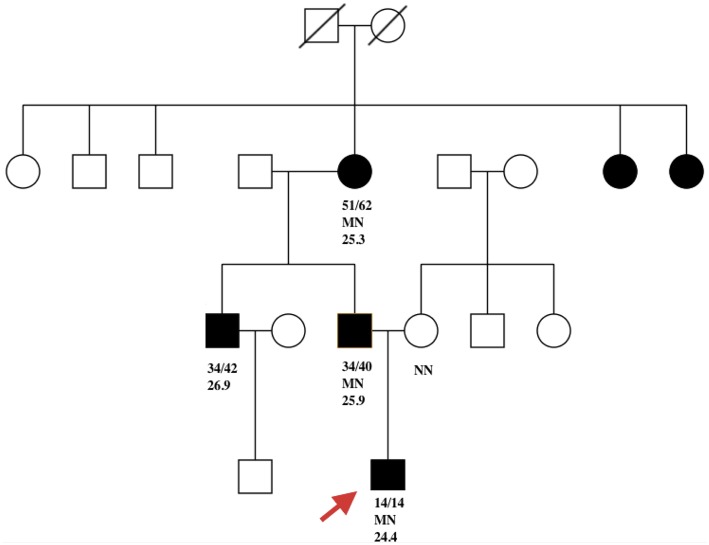
Filled black symbols represent individuals with diabetes. Age of diabetes onset (a) and present age (b) are represented as a/b. MN represents PDX1 c. 463C>A heterozygous mutation and NN represents the wild-type PDX1 gene. The remaining number represents BMI. Red arrow indicates the proband.

The father of the proband was found to be hyperglycemic (FBG 10 mmol/l) at the age of 34. His BMI was 25.9 kg/m^2^ at diagnosis. Glucose was controlled with metformin 0.25 g TID. His FBG is 7.2 mmol/L and 2hPG 8.9 mmol/L, with FCP 0.99 ng/ml and 2hCP 2.6 ng/ml. Both serum amylase and lipase were within the normal range. The proband's grandmother was diagnosed with diabetes at 51 years of age (BMI 25.3 kg/m^2^) and is now being treated with metformin. No test results for pancreatic enzymes and C-peptide were available. Diabetes was also diagnosed in the proband's uncle, who was in his thirties (BMI 26.9 kg/m^2^) and declined genetic testing. Two sisters of the proband's grandmother were also overweight diabetes, however no details of clinical features and genetic results were available (Figure 1).

**Figure 2:**
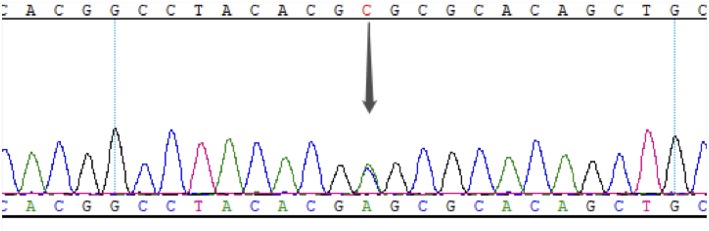
Results of Sanger sequencing of exon 2 in PDX1 of the proband. The gray arrow indicates the c. 463C>A heterozygous mutation.

This study was approved by the Ethics Committee of PUMCH, and informed consent was obtained from the proband and his family members. The proband underwent whole-exome detection by next-generation sequencing (NGS), which revealed a novel heterozygous missense mutation (NM_000209; c.463C>A) in the coding region, exon 2, of the PDX1 gene. This mutation was then verified by Sanger sequencing (Figure 2).

The father, mother and grandmother underwent Sanger sequencing for the target gene, and the same mutation was also detected in his father and grandmother. This mutation leads to a change in the amino acid sequence (p. Arg155Ser). The variant detected was not found in the 1000 Genomes and Exome Sequencing Project (ESP), and allele frequencies in the Exome Aggregation Consortium (ExAC) were 9.593 × 10^−5^. Both Mutationtaster (http://www.mutationtaster.org) and Polyphen-2 (http://genetics.bwh.harvard.edu/pph2/) predicted this variant to be pathogenic. Based on the 2015 guidelines developed by the American College of Medical Genetics and Genomics (ACMG) (7) for the classification of pathogenic or likely pathogenic variants, the novel PDX1 mutation identified in this family was considered “likely pathogenic,” based on the evidence framework of PM1 (located in a mutational hot spot and/or critical and well-established functional domain without benign variation), PM2 (absent from controls in ESP, 1000 Genomes, or ExAC), PP1 (cosegregation with disease in multiple affected family members in a gene definitively known to causes the disease), and PP3 (multiple lines of computational evidence support a deleterious effect on the gene or gene product). A heterozygous mutation of HNF1α (NM_000545, c.1107+9C>G) was detected by NGS, which was considered BS1 based on ACMG. No other diabetes-related genes were found in the proband.

## Discussion

MODY4 is caused by a mutation in the PDX1 gene (OMIM: 600733), which was first reported by Stoffers et al. (4). Herein, we report a MODY4 family with a missense mutation in exon 2 of the PDX1 gene c.463C>A (p.Arg155Ser). To the best of our knowledge, this variant has been detected for the first time and is the first MODY4 pedigree in China.

In the pedigree of our report, all affected family members were overweight at diagnosis suggestive of T2D. However, the low level of FCP in his father indicated pancreatic endocrine dysfunction. Moreover, although the proband was overweight, no hyperinsulinemia was present. We suggest that the normal levels of FCP and 2hCP are most likely a consequence of obesity. Similarly, Fajans et al. (8) studied the genetic and clinical features of diabetic subjects in a five-generation Michigan-Kentucky pedigree and found that both MODY4 and T2D were associated with obesity and hyperinsulinemia. The author suggested that there was at least a partial compensatory response in these subjects. In fact, obesity and obesity-induced hyperinsulinemia have been observed in MODY1, MODY3, MODY4, and MODY6 (9–11). Obese MODY and T2D are phenotypically indistinguishable and can only be differentiated by genetic studies. Therefore, multigenerational obese diabetic subjects should not be excluded from genetic tests.

PDX1 appears to be a key regulator of islet peptide hormone expression and is also responsible for the development of the pancreas. Six families with heterozygous PDX1 variants related to MODY have been reported (4, 8, 12–15) without the exocrine function described in these studies. Pancreatic agenesis has only been reported in PDX1 mutated homozygosity (16) or compound heterozygosity (6). However, Caetano et al. (17) recently described dorsal pancreatic agenesis in two Brazilian patients with MODY4, and one of them also had exocrine insufficiency. In our study, although no pancreatic agenesis was detected, the proband suffered from abdominal pain, and his serum lipase was slightly low, which characterizes exocrine pancreatic insufficiency. His father did not present with low pancreatic enzymes.

**Figure 3:**
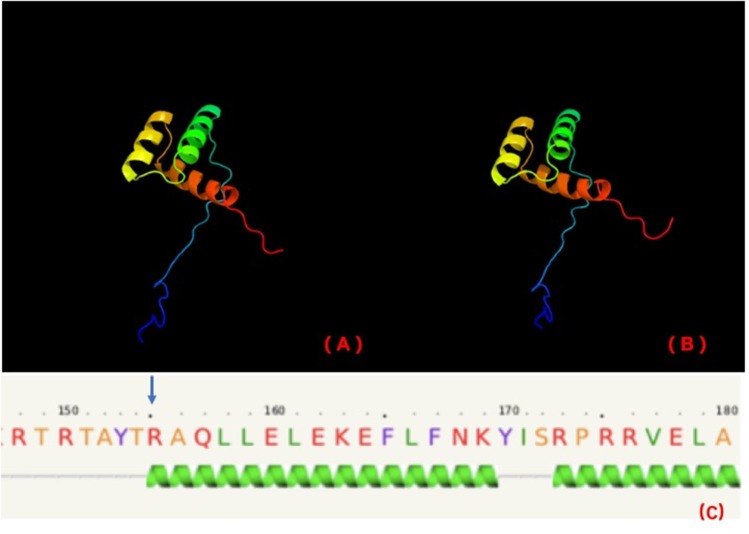
PHYRE2 predicts no difference in the structure of wild-type **(A)** and mutation **(B)**. The 155th amino acid (blue arrow) is predicted to be in the first helix of IPF1 of wild-type **(C)**.

Stoffers et al. (18) suggested that the mechanism of diabetes in individuals with this mutation may be not only reduced gene dosage but also a dominant-negative inhibition of transcription of the insulin gene and other beta cell-specific genes regulated by the mutant IPF1. The variant in our cases is in the homeobox domain of IPF1 (http://www.ebi.ac.uk/interpro/). The homeobox domain is well-conserved in many animals. It binds DNA through a helix-turn-helix (HTH) structure. The HTH motif is characterized by two alpha helices, which make intimate contacts with the DNA and are joined by a short turn. PHYRE2 (http://www.sbg.bio.ic.ac.uk/~phyre2/html/page.cgi?id=index) predicts that this variant is in the first helix of the homeobox (Figure 3).

As the first helix helps to stabilize the structure in a homeobox domain, we further investigated the stability of this variant by MUpro software (http://mupro.proteomics.ics.uci.edu), which indicated that this variant leads to a decrease in stability (confidence score = −0.965). Therefore, we suspect that this variant is at least partly pathogenic because of the stability decrease of HTH, which may affect IPF1 binding to the *INS* gene. However, functional tests *in vivo* and *vitro* are necessary to establish pathogenicity.

In conclusion, this is the first report of MODY4 in a Chinese family, and we report a novel PDX1 mutation. Pancreatic endocrine insufficiency may be unmasked in PDX1-deficient diabetes with obesity, and genetic testing should be carried out in young early-onset obese diabetic patients with a family history of diabetes.

## Data Availability

The data for this study can be found in the NCBI Sequence Read Archive under accession number SRR9613620 (https://dataview.ncbi.nlm.nih.gov/object/SRR9613620).

## Ethics Statement

This study was approved by the Ethics Committee of PUMCH. All patients provided written informed consent to participate in the study, which was conducted in accordance with the Declaration of Helsinki and Good Clinical Practice. Written informed consent was obtained from the proband and his father for the publication of this case report.

## Author Contributions

MD collected the clinical data and wrote the manuscript. LZ and TW collected the clinical data and summarized the relevant literature. All the work was performed under the instructions of XX. All the authors have contributed significantly.

### Conflict of Interest Statement

The authors declare that the research was conducted in the absence of any commercial or financial relationships that could be construed as a potential conflict of interest.
